# *Walk With Me*: reducing harm and confronting the toxic drug poisoning crisis in small British Columbia cities through community engaged research

**DOI:** 10.1186/s12954-024-01022-w

**Published:** 2024-05-31

**Authors:** Trevor Wideman, Sharon Karsten, The Walk With Me Team, Elder Barb Whyte, Kathleen Haggith, Christopher Hauschildt, Sophia Katsanikakis, Sharon Karsten, Andrew Mark, Caresse Nadeau, Trevor Wideman

**Affiliations:** Faculty of Education, Vancouver Island University, 900 Fifth Street, Nanaimo, BC V9R 5S5 Canada

## Abstract

In an era of escalating and intersectional crises, the toxic drug poisoning crisis stands out as a devastating and persistent phenomenon. Where we write from in British Columbia (BC), Canada, over 13,000 deaths have occurred in the eight years since the toxic drug poisoning crisis was declared a provincial health emergency. While many of these deaths have occurred in large urban centres, smaller rural communities in British Columbia are also grappling with the profound impacts of the toxic drug poisoning crisis and are struggling to provide adequate support for their vulnerable populations. In response to these challenges, the *Walk With Me* research project has emerged in the Comox Valley of Vancouver Island, BC, employing community-engaged methodologies grounded in pluralist knowledge production. Walk With Me seeks to understand the unique manifestations of the toxic drug poisoning crisis in small communities, identifying local harm reduction interventions that can foster community resilience, and aiming to catalyze sustainable change by amplifying the voices of those directly affected by the crisis to advocate for policy changes. This paper outlines the conceptual and methodological underpinnings of the *Walk With Me* project as a harm reduction initiative, which holds community partnerships and diverse ways of knowing at its heart. It presents the community-engaged research framework used by the project to address overlapping health and social crises, offering practical examples of its application in various research projects across sites and organizations. The paper concludes with a reflection on the impacts of *Walk With Me* to date, highlighting the lessons learned, challenges encountered, and opportunities for future research and action. Overall, this article captures the urgent need for community-engaged approaches to address the toxic drug poisoning crisis and other multidimensional crises facing society, particularly in smaller and rural communities, underscoring the potential for meaningful change through collaborative, grassroots efforts.

## Introduction

We live in a time of rapidly intensifying and overlapping crises. Lack of housing, disconnection, and trauma are just a few of the many societal issues that have, and are, compounding within a perfect storm where the toxic drug poisoning crisis (TDPC) continues to rage.^1^ In the Province of British Columbia (BC), Canada, where our research is located, 2546 deaths occurred in 2023, a 256% increase over the death count in 2016 (996) when toxic drug poisoning was labelled a provincial health emergency [6]. Importantly, smaller, rural communities in the province have not escaped the effects of this crisis, despite being less visible in relation to predominant urban discourses. And despite variations in their historical background, geographical location, and economic structure, small communities face a common dilemma in addressing the health and social welfare needs of their most at-risk residents. These communities struggle to provide the level of accessible social, health, and economic support that is readily available in larger urban centers. Those who are socially and economically marginalized or require special considerations often find themselves underserved and overlooked, while service providers face challenges in reaching these individuals [7, 8]. What has become apparent within this complex milieu is that dominant positivist and biomedical research and health care practices, while providing a strong evidence base for policy change and population health intervention, can struggle to address the pluralism and relationality of the poly-crises that are being faced by society at large. It can also be difficult to analyze and address the unique dynamics of the crisis as it unfolds in smaller communities using biomedical methods [9–11]. It is within this context that our community-engaged research project entitled *Walk With Me*, has emerged.

*Walk With Me*, located in the Comox Valley of Vancouver Island, BC, is committed to pluralist modes of knowledge production that are embedded in trusting community partnerships, with respect to the multiple ways of knowing that exist in community [see 12]. Our focus is on the impacts of the TDPC in smaller communities in British Columbia, particularly on Vancouver Island, which (despite small variations between cities), are being deeply and increasingly affected by a toxic opioid supply contaminated primarily by fentanyl and analogues, xylazine, and benzodiazepines, as well as by increased access to a highly potent supply of methamphetamines [6, 13].^2^,^3^ Our project asks: “how can community-engaged research help save lives, reduce harm, improve social cohesion, and create systems change for populations facing the toxic drug poisoning and related crises first-hand in small and rural communities?” Our goal is to comprehend the distinctive unfolding of this crisis in British Columbia's small communities and bring attention to narratives of adversity and strength that exist within them. By acknowledging and sharing the stories of those most impacted by the toxic drug poisoning and related crises, and by advocating for policy changes originating from those directly impacted by such crises, we aspire to cultivate an environment conducive to lasting change.

In what follows, we discuss our research processes to date, first by laying out the conceptual foundations of the *Walk With Me* project. We then provide a broad outline of our methods as a framework for action to address overlapping health and social crises in BC and beyond. We continue by providing brief examples of how our conceptual framework and methods have been deployed in practice, “on the ground,” in our various projects. We conclude with a discussion of the impacts of *Walk With Me*, reflecting on the lessons we have learned through the research process, the challenges of replicability and expansion, and opportunities for the future.

## Epistemological foundations of *Walk With Me*

### Community-Engaged Research (CER)

"When I think about what community engaged research is, I think about contesting [extractive research]. I think about what it means to have reciprocity between researchers and communities. And I also think about ways to remove that line between the researcher and community and acknowledge the wisdom communities innately have to understand themselves. And that wisdom has great value if it's brought forward to make change."—Sharon, *Walk With Me* team member.^4^

The *Walk With Me* project, while initiated as an urgent response to the TDPC in the Comox Valley of BC, where over 175 lives have been lost to toxic drugs (primarily contaminated by fentanyl and analogues) since 2016 [6]. Our project is rooted in an existing body of community-engaged research practices that have been evolving across academic disciplines over the past thirty years [16].^5^ CER (along with its core principles of reciprocity, relationship and capacity building, community expertise, democratization of knowledge, and the blurring of disciplinary boundaries) has become recognized as a powerful tool for generating social and political change in the context of multiple and overlapping crises [17]. CER positions marginalized and often-overlooked community actors as experts on their own living environments, with significant expertise to contribute to policy discussions, equal to state actors and others who normally have a ‘seat at the table’ [5, 17]. Such research is grounded in collaboration and engagement with those who are most affected by community crises (in our case, the TDPC, and its overlap with poverty, homelessness, and other ‘wicked’ societal issues), and seeks to ensure their equitable participation, with the goal of producing research results that can address the TDPC and bring awareness and action for change [5, 18, 19].

CER principles in research have been used extensively in work with Indigenous communities, for many of whom ‘research’ is a problematic word due to its extractive and colonial connotations [20]. CER has also become increasingly utilized in work involving people with lived and living experience of the toxic drug poisoning crisis (PWLLE^6^), whose lived expertise has often been ignored within research [25]. Crucially for our work in British Columbia, these two groups are not mutually exclusive, with many PWLLE identifying as Indigenous, and vice versa [26]. Thus, CER approaches have been central to our work as they seek to counter the extractive tendencies of dominant research practices and place the community as equal partner in the process [see 27]. Indeed, as team member Christopher, who identifies as a PWLLE, shares, our team takes on “a great deal of responsibility to ensure that the voice and the work of the research […] doesn't exist to extract from community but rather to serve it.” As other researchers have noted, CER promotes equity by involving communities as Peer researchers who participate in decision-making, co-design the research and set priorities, and decide how the results will be presented to the public and policymakers [18]. Moreover, CER is critically important in advancing decolonial work that walks with Indigenous Peers and communities in respectful, reciprocal ways to “reflect and honour [their] experience” and lead to robust research outcomes that may not have been possible without their involvement [18, 28, 29]. It can also empower communities in finding positive solutions to pressing social problems at the local level and enhance social and emotional well-being, while helping to avoid what Tuck refers to as “damage-centred research,” or, research which highlights community brokenness as its underlying theory of change [30, 31]. Such assertions also hold beyond the Canadian context. Community-engaged harm reduction research emerging from Australia has shown the limitations of biomedical research approaches in work with Indigenous peoples living in smaller/rural communities, showing the strengths of communities and demonstrating how cultural safety and connection are key to empowering and supporting the well-being of Indigenous PWLLE dealing with mental health and addictions challenges [31, 32].

Key here is that CER works to both produce knowledge and create change in collaboration with communities [27, 33, 34]. Unsurprisingly, then, CER principles often sit uncomfortably alongside dominant biomedical research approaches and associated ethical protocols, which, while seeking to limit risks and damage to individuals and institutions, can at times limit opportunities for collective agency/expression and structural/societal change [9, 27,see 35, 36]. While *Walk With Me* does not view CER in opposition to biomedical research, and while our work relies on data and results produced though biomedical research as a way of demonstrating the enormity of the TDPC, the ethical expectations around researcher distance/objectivity and participant confidentiality that shape biomedical research can hamper our ability to form trusting relationships with participants or recognize their contributions to research in a way that meaningfully reflects their desires (27,see 37,38). Moreover, the expectations of reliability and replicability that are built into deductive modes of research are difficult to meet (and even undesirable) within qualitative CER research projects such as ours that are grounded in place and concerned with driving community change at the grassroots level. Even beyond these questions of research design and participant engagement, biomedical methods and studies may also be limited in terms of the kinds of changes they are able to drive, and the ways that they are able to impact the TDPC on the ground. Evidence from BC suggests that the objective and value-neutral stance taken by epidemiological and biomedical studies is often more suited to assessing the effects of the TDPC rather than excavating the structural or locally differentiated political and social factors that shape addiction, engagement with toxic drugs, and harm reduction programs [see 39, 40]. Other studies from BC have also shown how long-term use of biomedical methods to research marginalized populations in place has led to research fatigue and lack of trust within, and stigmatization of, certain communities, while also hampering the ability of researchers to collect data, enact programs, and make meaningful policy recommendations for addressing the TDPC that reflect community desires [25, 39]. On the other hand, trust between researchers and participants, as established through CER, has been shown to produce robust research results that can produce promising and grounded policy recommendations around sensitive harm reduction topics, such as the BC government's recent provision of a safer supply of drugs [3, 18, 41].

That said, CER approaches are far from perfect. Researchers have catalogued a host of challenges that can affect CER projects, including funding issues, systemic institutional barriers, academic timelines, ethical challenges, questions around data ownership and benefits of research results, and whether research is truly driven by community [42]. In addition, principles are often applied inconsistently, and further, collaborations can be difficult to sustain [25, 43]. For example, some individuals can be difficult to maintain engagement with due to transience or lack of interest in a project, and some groups may cease involvement with a project because of shifting internal politics or systemic pressures and changes (social, financial, political, or otherwise) [43]. Yet we believe CER represents a sound epistemological basis from which to ground our work in generating knowledge and activating transformative change in community, institutional, and government settings. We honour the growth and potential of CER as a pluralist mode of research which our team uses to address complex and multi-faceted challenges and promote community well-being, social bonds, relationships, and interconnectivity. At the heart of our work (which proceeds based on invitation from, the guidance of, and strong relationships with community partners) is a commitment to creative and cultural practices, in particular, arts-based practices that are integrated with ***sharing circles*** guided by an Indigenous Elder/Knowledge Keeper. Indeed, as we discuss next, the circle has emerged as an important metaphor, organizing principle, and epistemological framework that anchors our community-engaged work.

### The circle

"Creating from the mind I set goals and reach for them, work towards them, individual minds stimulated by their individual thoughts are sometimes difficult to blend. In creating from the heart, you simply open your heart, see what comes forth. When you sit in a circle with many hearts and work to create something, what is that something? It’s something that comes from hearts collaborating for creation"—a teaching on the power of the sharing circle, gifted to us by Pentlatch Elder Barb Whyte, *Walk With Me* team member.

The circle, as a spiritual teaching and as community-engaged research method, permeates everything that *Walk With Me* does. Regarding the spiritual pieces, the circle teaching, and its associated practice, the sharing circle, was introduced and gifted to the *Walk With Me* team by Pentlatch First Nation Elder/Knowledge Keeper Barb Whyte, who provides the team with ongoing guidance on how to conduct community sharing circles in ways that align specifically with Pentlatch Coast Salish teachings, and with respect for the circle practices of Elders and Knowledge Keepers in the territories in which we work. The circle also describes how relationships, experiences, and knowledges are made active within the project. It is grounded in the key principles of equality, respect, and heartfelt communication, among other principles, which means that we take turns sharing, moving to the left (signalling our intent to speak from the heart), while listening closely to and honouring others when they speak. In taking turns speaking and listening, participants are afforded time to interpret and process the words and experiences of those around them. We use the circle practice in every aspect of our work: from everyday team meetings to our research fieldwork (where participants share stories and reflect), to the circle dialogues that we hold with community leaders and participants after our public events. The power of the circle is that it is both a spiritual practice and a way of working in community. As Elder Barb shared:

“The circle respects everybody that is sitting in it and gives everybody a voice […] it empowers those individuals that haven't been given a voice before. And that empowerment helps to lift people up in the community in a gentle way […] and brings up awareness for them as well.”

**Fig. 1:**
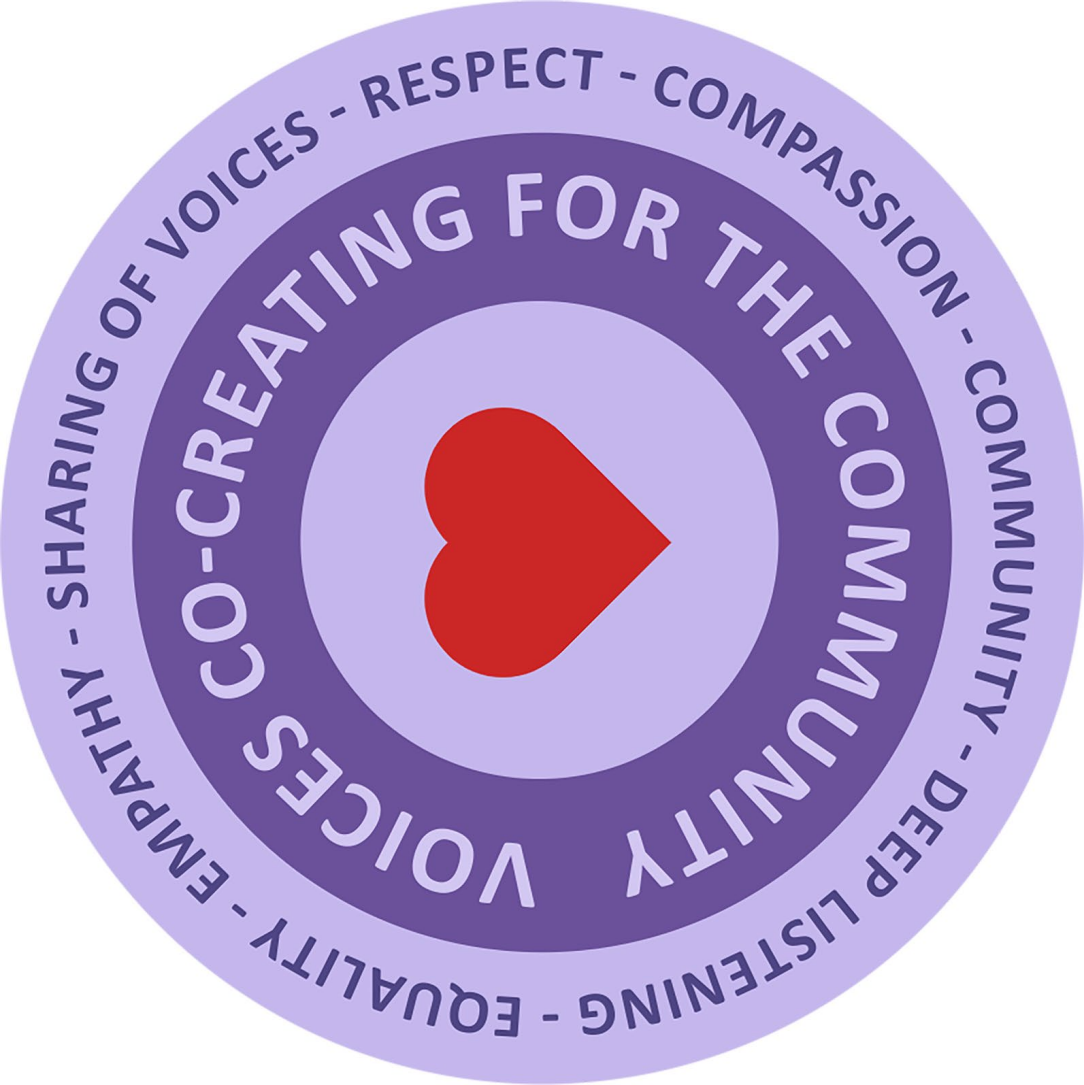
The circle as a spiritual practice (image based on teachings by Elder Barb Whyte, design by Caresse Nadeau

As a spiritual practice, the circle provides our team with a holistic foundation and dynamic approach to community engagement in that it can bring diverse and sometimes divergent voices together in respectful dialogue with the potential for positive and unanticipated outcomes (see Fig. 1).

**Fig. 2:**
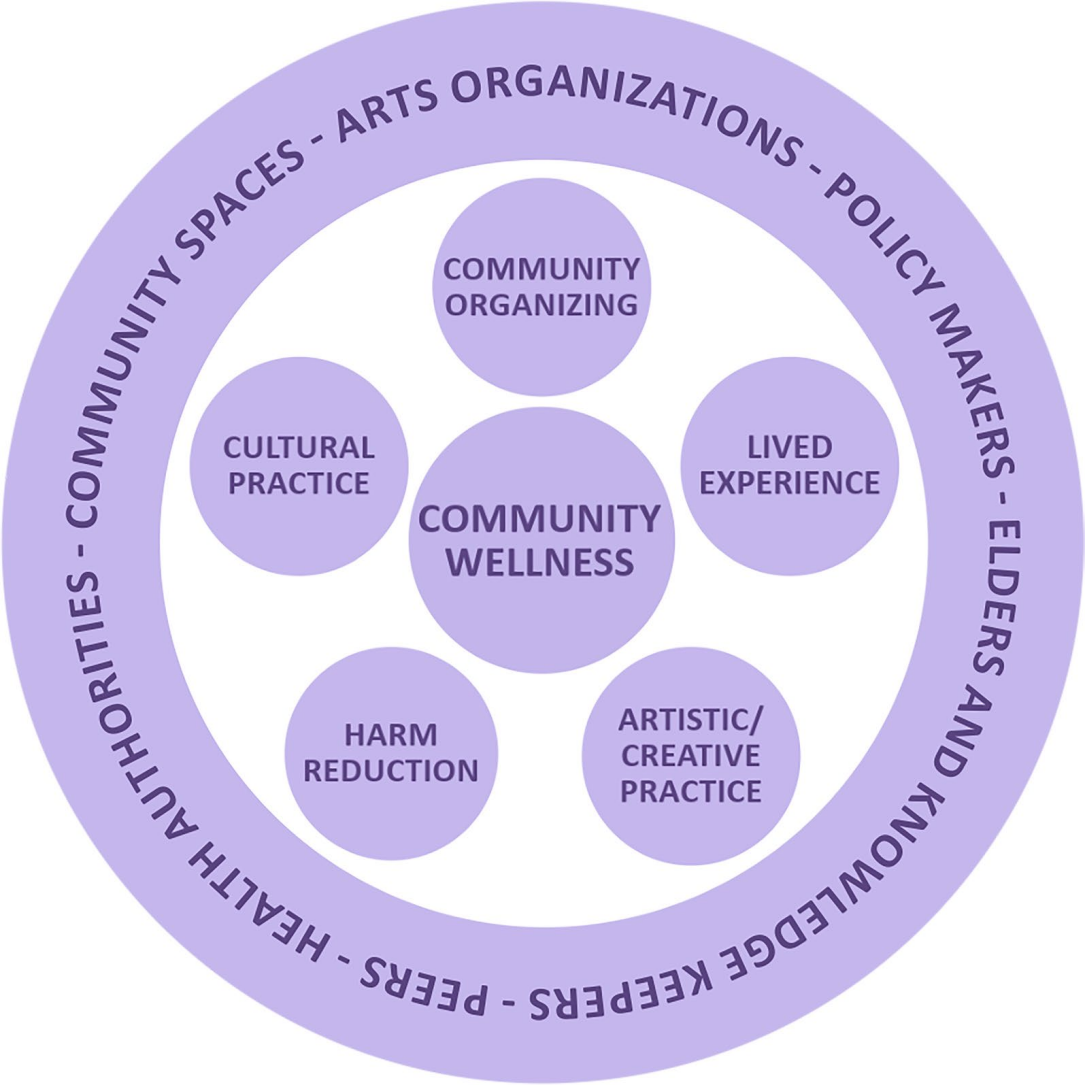
The circle as a community-engaged research practice (design by Caresse Nadeau)

In addition to being a spiritual practice, the circle operates as a mode of community organizing within *Walk With Me*. Here, five key domains (or, types of knowledge and experience) have emerged that comprise a holistic vision of the circle in community-engaged research: as a cultural practice, a creative practice, as lived experience, as harm reduction, and as a site for community organizing (see Fig. 2). While we have described the intricacies of these domains in detail elsewhere [5, 44], we outline them briefly here to demonstrate how they provide grounding for *Walk With Me.*

The first domain of the circle, **research as a cultural practice**, is arguably the most important of the five as it acknowledges and honours the Indigenous cultural practices, and concomitant knowledges and epistemologies, that have been imparted to us by Elders and Knowledge Keepers. Indigenous epistemologies and practices have long been recognized as a robust, community-engaged, and place-based alternative to the rationalist approaches that have long dominated research with (and often on) Indigenous peoples [20, 45]. Cultural practice is a critical component of our work not only because we have Indigenous team leaders, but because the TDPC in BC disproportionately affects Indigenous peoples—despite making up only 3.3% of the province’s population, 16% of toxic drug poisoning deaths in 2022 were Indigenous [46]. In addition, many of our participants identify as Indigenous, and the unceded land on which we walk is scarred by the ongoing violence of settler colonialism, and the institutions, structures, and logics that hold it in place [see 47–49]. Recognizing our team’s power and privilege, and recognizing that culture and circle practices look different in every community, we also hold cultural safety as a key component of care within our community-engaged practice, with the goal of easing participants’ feelings of stigma and powerlessness [see 50]. As Elder Barb notes:

"each community carries their own culture. [...] The leaders guide the culture in that community [...] by sharing the gifts and the knowledge that they have through their spoken word that they share in the circle. Cultural teachings are often passed down intergenerationally, to ones chosen by a leader to serve the community. We all have gifts, we all have a different lens in how we share our culture. By deep listening in the circle, we recognize those gifts and can carry them further once the words are spoken. To have respect for each voice in the circle allows that person's gifts to come out and evolve."

With this in mind, and considering how the cultural assumptions underpinning academic research have long done great harm to Indigenous peoples, our team has worked to embed cultural practices and cultural safety into our work, with the circle practice at the core. Indeed, as team member Sharon states: “the circle is a big part, if not the foundation of, cultural safety […] which resides in respect and relationship.” We are inspired by the work of Indigenous scholar Shawn Wilson, and see research as an act of relationship building and ceremony that entails deep accountability to the communities with whom we are engaged [51].

**Research as a creative practice** forms the second domain of the circle, and it speaks to *Walk With Me*’s commitment to artistic practice as a mode of expression and a vector for the flattening of hierarchies between researchers and participants. Here, artists and arts institutions hold a key role in advancing our work and activating creative modes of sharing, where participants can express themselves within the project in ways they feel most comfortable (e.g. songs, stories, photos, prints). There is a robust scholarly tradition of arts-based methods being used in qualitative community-engaged research, particularly with vulnerable populations [52], and we stand on these foundations as we work with participants to articulate their lived experience in ways that defy standardized reporting mechanisms (surveys, structured interviews). While there can be significant challenges in taking on arts-based methods, including the constant evaluation and renegotiation of research practices, and the need to continually redefine what constitutes “rigorous” research [53, 54], in our work, arts-based methods are crucial as they operate as an emergent and organic practice which shifts depending on the setting and the participants. In our relational use of creative methods, we make room for unforeseen events to occur and unanticipated research results to materialize [see 55].

The third domain of the circle is marked by **research as a lived experience.** *Walk With Me* places PWLLE of the toxic drug poisoning crisis at the forefront of what we do. In doing so, we respond to and build on a grassroots demand from marginalized communities who have called for their active inclusion in impactful decisions that affect their wellbeing, articulated under the banner of “nothing about us without us” [56, 57]. Since this demand was first placed, researchers, health and social services providers, and policy development actors have slowly been responding and making efforts to include those with lived experience in their work [43]. That said, there remain challenges with promoting the equitable inclusion of PWLLE into research and health care settings, where significant power differentials remain. In the worst cases, those with lived experience are tokenized in research and framed as ‘subjects’ rather than ‘participants’ or ‘collaborators,’ a process that devalues their experiences and fails to challenge dominant research frameworks [43, 58]. As *Walk With Me* team member and PWLLE Christopher points out, "Peers [need to be] on the research team so that those in a disadvantaged position are informing [research] decisions.” We remain steadfast in our conviction that relationships with those with lived experience need to be at the centre of everything we do—to challenge the individualist status quo, pave the way for new forms of collective research, and gain new knowledge that can help stop the harms associated with the TDPC.

Building on this third domain, the fourth domain of our circle practice frames **research as harm reduction.** Harm reduction, as we see it, refers to a movement of people working to achieve social equity for those with lived experience of the TDPC, while taking a humanistic (rather than punitive) approach to policy and service provision [see 59]. For those working on the front lines of the TDPC, this domain of community-engaged research may seem obvious: if research is truly committed to involving people with lived experience, then it must meet them “where they are” and work to reduce the harm being done to them. However, this is often more easily said than done. Those working in harm reduction with PWLLE (both at a grassroots and academic level) understand that harms are not isolated—they are overlapping and highly complex, and can have intense impacts on the well-being of individuals and communities at large [see 60]. Moreover, such complexity is difficult to comprehend, let alone address, solely through quantitative methods of research and reporting [see 43]. Yet in deploying community-engaged research as a practice of harm reduction, our collaborative work aims to uncover layers of complexity by centring the wisdom and humanity of those at the heart of the TDPC. We strive to listen deeply and honour people in the moment. We take a humanistic approach to addressing the TDPC through community care, respect, and empowerment.

The fifth and final domain of the circle we call **research as community organizing.** This process involves activating and extending our existing networks and collaborations as a mode of community-engaged research. Community organizing, as an activist practice, involves weaving together and empowering networks of people to work collectively to address social issues of common concern, with the goal of generating systems and policy change [61]. For *Walk With Me,* the process of “scaling up” is key to our work: we do research at the grassroots scale to understand complex local issues, while deploying these learnings in pursuit of structural change at the policy level. While there can be significant challenges in breaking down siloes within community to address acute crises, including fear of retribution from some individuals or organizations, the failure of some potential allies to ‘come on board,’ and the possibility of upsetting funders [62], in our experience the achievements and benefits of community organizing have far outweighed the challenges and failures. In taking on research as a practice of community organizing, our networks have expanded, our human and financial resources have grown, and local knowledge has been transmitted outwards to a wide variety of community and policy actors.

Key here is that these five domains of the community-engaged research circle (cultural practice, creative practice, lived experience, harm reduction, and community organizing) are not mutually exclusive. They overlap and co-constitute each other within a complex, pluralist framework to shape our research work in community, and aid us as we work to translate diverse knowledges and experiences between Peers, community leaders, academic experts, and health-care providers. In what follows, we describe our methods. We describe the project’s primary method of cultural mapping and show how our methodological stance reflects each domain of the circle.

## Cultural Mapping: How we do what we do

*Walk With Me*’s methods are rooted in practices of *cultural mapping*, which, at its core, refers to a process of deep storytelling [63]. Emerging from Indigenous communities and community development programs in the early 1990s, cultural mapping has grown over the past 30 years from a framework for capturing local “intangible heritage” and demonstrating its value on an international stage [see 64, 65], to an instrument of collective knowledge building, communal expression, empowerment, and community identity formation [66]. Cultural mapping, as an action-oriented CER methodology, is important for our work in two ways: first, it can help “make visible” the first hand lived experiences of those experiencing crises in a community; and second, it can identify and weave connections between diverse actors in a community, with the goal of extending important insights articulated by people “on the ground” into the policy realm and generating systems change [67, 68]. Within a cultural mapping process, the “map” is both literal and figurative. For example, it might be a graphic representation that makes the intangible assets and experiences of a community visible, or it might refer to the process of gathering people together to build and strengthen a community network (68, see 69). Crucially, cultural mapping is not just about describing that which exists, but it is about speaking truth back to power and fostering deeper understanding about people’s lived realities [67].

In *Walk With Me,* cultural mapping occurs primarily through a draw-talk protocol. Similar to (and building on) other participatory action art-based methods such as photovoice or community mapping, where participants are invited to produce a piece of visual material that can be used as a basis for group discussion, analysis, and presentation to policymakers [see 70–72], the draw-talk protocol allows participants to ‘sketch out’ their lived experience and subsequently speak to their drawings in semi-structured interviews conducted with members of the *Walk With Me* team. This knowledge is then translated back to the community and to policymakers with the goal of generating systems change. This methodology is adaptive and responsive to the needs and preferences of participants, who at times use music or photography in addition to drawing to articulate their lived experiences.

*Walk With Me’s* cultural mapping process often takes place over a sustained period (1 year+) within a given community. The process is guided by, and exists in reciprocal dialogue with, the five domains of the circle noted above (cultural practice, creative practice, lived experience, harm reduction, community organizing), starting at a small scale at the individual or group level, and rippling outwards to the regional and provincial level (see Table 1). Our work is preceded by a period of relationship development and deep listening in community, including with cultural leaders, service providers, Peers, artists, and others, to understand local needs and establish protocols for the work to come. Understanding nourishment as a process of community building, we provide partners and participants with food (along with various other forms of support) throughout every phase of the work.

**Table 1:** Walk with Me’s cultural mapping process

Research stage	Activity	Active epistemological domains
*Pre-research*	Relationship Building Deep Listening Establishing Formal Partnerships	Cultural Practice Community Organizing
*Stage 1: Insight/Story Sharing*	Visual Art Audio Recording Sharing Circles	Cultural Practice Creative Practice Lived Experience Harm Reduction
*Stage 2: Community Dissemination*	Story Walks	Creative Practice Lived Experience Community Organizing
*Stage 3: Community Dialogue*	Sharing Circles	Cultural Practice Lived Experience Community Organizing
*Stage 4: Policy Reporting and Advocacy*	Research Reports Academic Publications Media Engagement Non-traditional creative formats (zine, podcast)	Cultural Practice Creative Practice Lived Experience Harm Reduction
*Post-research*	Maintaining Connections Following up on Recommendations Supporting Legacy Projects	Cultural Practice Harm Reduction Community Organizing

Having established relationships and laid the groundwork for the project; the work proceeds according to a four-stage process. ***Stage 1, Insight/Story Sharing,*** includes the draw-talk protocol described above, using artmaking and audio recording as ways of gathering insights from PWLLE, their family members, and front-line health workers who are grappling with the TDPC firsthand. ***Stage 2, Community Dissemination***, is the first step of knowledge translation and it involves the creation of curated art exhibitions and “story walks,” where the public is invited to walk and listen to the stories that have been given to the project through a transmitting headset system—often walking through the parks, streets, and alleyways of the place that the stories refer to. ***Stage 3, Community Dialogue***, often happens immediately following the walk, where those participating in the walk are invited to sit in circle and process these stories collectively, guided by Pentlatch Coast Salish teachings and the teachings of Elders and Knowledge Keepers in the territories in which we work. ***Stage 4, Policy Reporting and Advocacy*,** involves the production and dissemination of research reports and other documentation aimed at affecting systems and policy change at multiple levels of decision-making, from the local and organizational to the provincial. At the conclusion of this process, *Walk With Me* endeavours to establish legacy projects in community with partner organizations who can support, maintain, and expand this work. Importantly, this process is iterative and circular. When a project is “complete,” we return to the beginning, working with partners and collaborators to generate new research offshoots and expand the work to different organizations and communities.

Cultural mapping, as situated within the five domains of the circle, has proven to be a robust and flexible community-engaged research method for *Walk With Me*. It has allowed us to build and strengthen community bonds in place, understand the needs and lived experiences of those on the front lines of the TDPC, and generate strong, policy-relevant research results that resonate with decisionmakers. In the following section, we draw on our various reports and outputs to show more specifically how our community-engaged research practice of cultural mapping has been utilized within our projects over the past four years.

## Cultural mapping and circular research in practice

*Walk With Me* was initiated in the Comox Valley of BC in 2019 by staff at the Comox Valley Art Gallery (of which the principal investigator was director at the time) as a response to the effects of the TDPC on community members involved with, and living near, the gallery. As much of the available information on the TDPC in BC addressed the situation in cities such as Vancouver, the project team^7^ sought to find new ways of understanding how smaller communities in the province were being affected by the TDPC, particularly in the Comox Valley. From this, the qualitative cultural mapping protocol described above was developed, and the project has expanded and evolved over subsequent years as the TDPC has grown.

Since its inception, the *Walk With Me* team has worked in partnership with five academic institutions and eight non-profit organizations. We have also built strong relationships with key actors in two regional health authorities and within the BC provincial government. The work is funded by eleven different local, provincial, and federal funding agencies. As a result of these connections and relationships, we have been able to grow the work over the past five years to encompass nine communities, primarily on Vancouver Island but also across BC. Our collaborations have, to date, culminated in six reports communicating research results and recommendations specific to the communities and organizations we have worked with. In the following, we outline some of these collaborations with a focus on how our cultural mapping protocols have been deployed in practical and place/organization-specific ways.

### a. Walk With Me: Comox Valley

*Walk With Me’s* initial research was developed in 2019 as a collaboration between university researchers, community organizers, and an advisory team of municipal officials, health providers, and Peers. With the goal of understanding the TDPC from a qualitative and humanistic lens, our team sought to develop an innovative, participatory, arts-based research model that could help generate create robust knowledge around the TDPC in small communities. What resulted was the first iteration of the cultural mapping process described in Sect.3.

The team began hosting research sessions with Peers at the Comox Valley Art Gallery. These sessions included food, and they began with a presentation on ethics and informed consent. Participants were invited to answer the research question “How has the toxic drug poisoning crisis impacted you and your community?” through drawing, writing, or speaking, and were given the chance to elaborate on their insights in an audio-recorded sharing circle. In each session, the research team adhered to trauma-informed practice and cultural safety protocols, with an Indigenous Elder being present at all times [50, 73]. Audio recordings were subsequently transcribed and analyzed using NVivo software. Data from these recordings were used to inform the creation of (1) experiential audio journeys known as “story walks,” aimed at a broad audience, and (2) policy reports, aimed at policymakers, both with the goal of building awareness of the TDPC in the Comox Valley to enact systems change.

During this initial two-year collaboration, *Walk With Me* held 32 public walks and sharing circles which engaged over 500 participants, including members of the general public, local government officials, community non-profit actors, health authority employees, and Peers. Major findings informed a 2021 report entitled *Walk With Me: Uncovering The Human Dimensions of the Toxic Drug Poisoning Crisis in Small B.C. Communities—Policy Report: Comox Valley* [21]. Informed directly by the insights of research participants and with an eye towards the social determinants of health [see 60], the report highlighted people’s lived and living experiences of the TDPC in the Comox Valley. Through this work, *Walk With Me* was able to engage with a broad spectrum of community actors and changemakers, and make key recommendations that have been followed up on by numerous agencies in the ensuing years (themes and recommendations from the report are outlined in Table 2).

**Table 2:** Walk With Me—project summaries

Research project	Knowledge user	Stages employed	Participants	Select themes	Select recommendations
a. Comox Valley (2019–21)	Community	1–4	PWLLE Harm Reduction Service Providers Decisionmakers General Public	Toxic Drugs Stigma Racism Intergenerational Trauma Cultural Knowledge and Safety Safer Supply Harm Reduction Recovery	Decriminalization Safe Supply Drug Testing Programs to Address Stigma and Racism Improvements to Overdose Prevention Site More Recovery Services Community Services Hub Leadership Opportunities for PWLLE Evaluation of the Existing Substance Use Support Network
b. Island Health (2021–22)	Organization	2–3	Health Care Workers	Honouring PWLLE Inclusion of PWLLE in Decision-making Need for Full-Spectrum Care Service Integration Overwhelming Caseloads	Strengthen Peer Leadership Reduce Stigma Close Care Gaps Humanizing PWLLE Reducing Caseloads Staff Education and Innovation
c. Comox Valley-Substance Use Support Network (2022)	Community/Organization	1, 4	PWLLE Harm Reduction Service Providers	Lack of Targeted Supports Gaps in Recovery Services Gaps in Harm Reduction Need for More Collaboration Need for Culturally Safe Services	Medical Detox Recovery Based Supportive Housing Service Hub Expand Harm Reduction Network Integration and Collaboration Cultural Safety Reducing Stigma
d. Campbell River (2021–23)	Community	1–4	PWLLE Harm Reduction Service Providers Peer Leaders	Structural Dynamics of the TDPC Violence Local System Failures Community Strengths Pathways to Wellness	Culturally Informed Services Safe Spaces Health Service Coordination Community Hub Improved Harm Reduction Services Education and Employment Services
e. North Island College (2022–23)	Organization	2–3	North Island College Staff, Faculty, and Students	Gaps in Harm Reduction Need for Culture Change Connecting Beyond Campus Transforming Curriculum	Expanding On-Campus Harm Reduction Harm Reduction Training for Instructors Safe Spaces to Address Stigma Connecting with Harm Reduction Community Developing Educational Opportunities for PWLLE Creating a Campus Harm Reduction Hub Developing Curriculum to address the TDPC

### b. Walk With Me: Island Health

Building on the successful engagements, connections, and research findings generated through the initial Comox Valley phase of the research, in 2021 *Walk With Me* entered into a research partnership with Island Health, the Vancouver Island regional health authority, to conduct a series of audio walks and research sessions with over 200 staff across Island Health sites (Campbell River, Comox Valley, Parksville) to answer the research question: *“How can Island Health better support people at the heart of the toxic drug poisoning crisis?”* These sessions took place from September 2021 through June 2022, and research results were compiled in the report *Walk With Me—Pathways Forward: Island Health and the Toxic Drug Poisoning Crisis* [22].

The research methods used within Island Health differed from our previous work in that we primarily employed stages 2 and 3 of our larger cultural mapping process. Sessions proceeded as follows: First, individual Island Health staff members were invited to participate in research sessions using an ethics-approved invitation letter. Those who accepted gathered to sit in circle and participate in a 40 min story walk. In circle, participants were oriented and led through informed consent and ethics procedures to prepare them for the walk, with an Elder present and cultural safety and trauma-informed protocols in place. The group walked along a predetermined route near the hospital site, listening to audio tracks that foregrounded the voices of PWLLE and which were compiled by the team during the previous Comox Valley phase of the project. Upon returning, participants were offered food and participated in a ~ 90 min facilitated and audio-recorded research circle where they were invited to answer the primary research question. Following the circle, responses were transcribed, coded, and analysed by the research team.

The modified cultural mapping research methods used by the team in this project created a safe and welcoming environment where staff were able to share ideas for systems change, foster community, and generate mutual understanding in a spirit of openness. Participants were inspired by the voices of PWLLE as they reflected on their expereinces during the walk. From these reflections emerged themes around honouring Peer leadership, filling acute gaps in the health care system, and unlearning negative and toxic practices, which generated six recommendations aimed at fostering organization-wide change (see Table 2). These recommendations were included in the report and have been taken up by Island Health as it strives to transform its response to the TDPC in more humanistic and less stigmatizing ways.

### c. Walk With Me: Comox Valley Substance Use Support Network—Gaps and Strengths Analysis

Responding to a key recommendation from the *Walk With Me: Comox Valley* research, in Spring 2022 our team set out to map and assess the network of substance use supports and services available to Peers in the Comox Valley. Included in our definition of Substance Use Support (SUS) network were the existing system of harm reduction, recovery, health, and mental health services, as well as upstream factors that influence people’s engagement with this system. The goals of this research were to look at the strengths and gaps within the existing support network, shine a light on local assets that might enhance the system, and produce recommendations that would sustain and grow the network over the long term.

As in the research with Island Health, a modified cultural mapping process was employed to assess the SUS network, this time utilizing the pre-research and stages 1 and 4 of the larger process. Participant recruitment proceeded through existing community relationships, public calls for participation, and snowball sampling. The team hosted 16 small group sessions that engaged with 59 PWLLE and 25 representatives of local service providers. After being oriented to the project, participants were led through a draw-talk protocol where they were invited to map or draw out the SUS network and speak to their experiences. After the mapping exercises were complete, groups sat in circle and participated in an audio-recorded focus group, where they could speak more deeply to the materials that they had produced in the session. Insights from these sessions informed the writing of a community report. Before the report was finalized, the team undertook a rigorous process of member checking, ensuring that participants were comfortable with how their voices were used in the report [74]. Participants were also given a chance to participate in a “peer-review” session where they could give feedback on a report draft, comments and suggestions from which were integrated before the report’s final release to the public. Qualitative findings in the report were bolstered by data collected through an anonymous online survey that was administered to 51 PWLLE in the Comox Valley, which provided a quantitative “snapshot” of the local SUS network in terms of access and quality of services.

Themes emerging from the cultural mapping and survey processes showed significant gaps combined with remarkable strengths in the Comox Valley SUS network, and from participant reflections came 11 recommendations (see Table 2). These recommendations have been key in generating improvements in the existing network of supports, while providing impetus for network expansion.

### d. Walk With Me: Campbell River

In Fall 2021, *Walk With Me* was invited by cultural leaders and service providers in Campbell River, BC to undertake a series of research sessions and story walks in response to a need for more information on the social impacts of the TDPC. Campbell River has, since 2016, seen a similar number of deaths from toxic drugs as the Comox Valley (~ 175), with an opioid supply increasingly tainted by fentanyl and benzodiazepenes [6, 13].^8^ Within this context, our initial sessions formed the foundation of a deep research relationship with Peers and their allies in the community which stretched throughout the COVID-19 pandemic through to Spring 2023. The research process in Campbell River entailed deep learning and strategic adjustments to our protocols as we sought ways to do research in a new community in a good way. Like our initial research in the Comox Valley, the cultural mapping process used to produce the Campbell River report encompassed all aspects of the larger process, from the pre-research phase of relationship building, to the current post-research phase in which we are maintaining our relationships within the community, following up on recommendations, and supporting legacy projects. Where the process differed was that we needed to slow down, build relationships, and check in with partners and participants frequently throughout the process to ensure that the work (1) was responsive to ever-changing community needs, (2) respected the protocols and territories of the Indigenous nations in Campbell River, and (3) honoured the vulnerability of participants who entrusted their words to us in the face of ongoing crisis and violence.

Our primary method of data collection in Campbell River involved the draw-talk protocol described above. Over 70 participants attended our recorded research sessions, each of which had Elders present and informed consent and cultural safety protocols in place. Research participants were invited to speak to their lived experience of the TDPC in Campbell River, and the impacts of the TDPC on the community at large. Participants were also asked how they would envision individual and community wellness, with the goal of creating improvements in the existing support network. The insights from these sessions were complemented by one-on-one interviews with key harm reduction actors in the community. Audio recordings of interview sessions were transcribed and coded thematically before being consolidated into the final report, which went through a rigorous process of member checking before its wider release.

Three years of research in Campbell River produced *Walk With Me’s* largest set of findings to date. Participants shared specific aspects of their experiences with the TDPC along five major themes, which generated a set of ten recommendations, primarily centred on the creation of new and expanded services, as well as improved coordination between existing services (see Table 2). Notably, some of the recommendations in the report are already being acted upon, even if their ultimate impact remains to be seen.

### a. Walk With Me: North Island College

In 2022 *Walk With Me* entered into a partnership with North Island College’s (NIC) Faculty of Health and Human Services and brought the project onto a community college campus. Evidence suggests that the TDPC is affecting college and university campuses in Canada in unprecedented ways, and NIC, which operates four campuses across North and Central Vancouver Island, is not immune to these trends [see 75–77]. Moreover, the campus is highly integrated into the health care system on Vancouver Island, and students who graduate from NIC programs often move directly into jobs in health, community care, and service organizations within the region. Building on conversations with NIC staff that had been occurring since 2019, and harnessing *Walk With Me*’s existing successes doing research within an institutional environment, the team partnered with NIC Health and Human Services with the intention of raising awareness of the TDPC, reducing stigma, and generating systems change among students preparing to enter the local health care workforce.

From March 2022 to March 2023, NIC students, faculty and staff participated in the research across 19 two-hour sessions. The methods employed comprised of stages 2 and 3 of our cultural mapping process. Having been oriented and given consent, participants took part in a story walk in which they listened to curated audio tracks featuring the voices of PWLLE. Upon completion, participants were invited to sit in circle and reflect on the story walk, with reference to the research question: *“how can NIC better-support people within its community at the heart of the toxic drug poisoning crisis?”* and *“how can NIC better support students entering a world where the crisis is raging?”* As they engaged in the circle, participants contributed to a reciprocal modality where they shared, listened, and learned from each other’s stories, insights, and lived experiences, with an Elder present and with reference to trauma-informed practice and cultural safety protocols. In contrast to research sessions in previous projects, the *Walk With Me* team noted a limitation in that interactions with students were brief, and response time was constrained. Yet regardless of this limitation, key insights were gleaned from participants and consolidated into a final report entitled *Equipping Changemakers: Uncovering North Island College’s potential to spur Culture, Community, and Systems Change in response to the Toxic Drug Crisis*.

NIC research participants recognized the magnitude of the TDPC and the ways in which it was affecting the Comox Valley. But more specifically, participants looked “inward” at the NIC campus and identified key areas in which harm reduction services could be developed and/or expanded for the benefit of the campus community and beyond, summarized in the phrase “we all matter.” Responses were coded across four major themes and resulted in seven recommendations (see Table 2), which were aimed at college leadership and the wider campus community to reduce harm, deaths, and stigma associated with the TDPC in Comox Valley and North Vancouver Island, while positioning NIC as a social innovator on the provincial and national stage.

## Discussion and Conclusion: Impacts, challenges, and opportunities

In its short lifespan, *Walk With Me* has taken on a key mediating role in building dialogues between disparate actors across Vancouver Island, and in communicating the lived and living experiences of people affected by the TDPC to the general public and to those that hold the power to make policy change. Many of the recommendations made in our five reports have been taken up by Vancouver Island agencies, governments, and organizations seeking to create meaningful societal change around the TDPC. Here we highlight three key instances where our recommendations have been acted upon (either directly or indirectly), recognizing that *Walk With Me* is but one actor within a larger community of harm reduction advocates working, in many cases, towards the same goals.

First, building on the learnings and recommendations from our first Comox Valley report (2021), and on institutional relationships we built in the region, we worked alongside various local health and human service organizations to assess the Comox Valley Substance Use Support Network, an analysis that was published in the *Walking Together* report (2023). This work, which drew on the voices of both Peers and local service providers, identified and demonstrated gaps in the local substance use support system, including stigma, lack of local recovery services (particularly detox and supportive housing), inadequate harm reduction services (especially around safer supply), poor collaboration and integration between existing services, and lack of culturally safe services for Indigenous PWLLE (see Table 2; 23). Recommendations emerging from this report included the establishment of local medical detox and supportive recovery housing, an integrated and networked service hub, expansion of existing harm reduction services, stigma reduction programs, and cultural safety training (see Table 2; 23). Second, these recommendations have already gained significant traction within the Comox Valley, having been incorporated into the Comox Valley Community Health Network’s Phase 2 Report [78]. In addition, a community-wide Collaborative has been developed by the Health Network, comprised of key stakeholders, and a series of action tables created with intent to move the recommendations forward. Specific recommendations gaining traction in the Comox Valley (in collaboration with other organizations such as Island Health) include (1) the development of a Community Health Services Hub located in the Comox Valley; (2) relocation/expansion of the Overdose Prevention Site in Courtenay; and (3) the creation of Peer Assisted Care Teams to help address concerns around stigma, cultural safety, and inclusivity within the local SUS network [78]. Finally, the recommendations listed above, in addition to those in the Island Health report (2021–2022) (see Table 2) have facilitated institutional transformations and led to closer integration between *Walk With Me* and Island Health. In particular, *Walk With Me* continues to run story walks and conduct research within Island Health sites with the goal of addressing stigma and creating awareness for change among health care workers around the TDPC. Our team is also collaborating with Island Health in the creation of a Harm Reduction Learning Health System model which highlights the perspectives and real-life encounters of Peers and is a key example of an innovative (and institutionally integrated) approach to addressing the TDPC [79]. A Learning Health System is one which affected communities, health care professionals, and researchers engage in a process of co-learning to identify gaps in evidence and create new knowledge, ultimately aiming to transform the system, decrease mortality rates, and enhance overall quality of life for those directly affected [80].

Yet as our team reflections and discussions have revealed, with these successes have come challenges. As we have continued to walk, we have learned important lessons from those we walk alongside. Given the community-engaged and place-based work that we do, we recognize that there are many important considerations as the project continues to grow, and the first challenge we have identified is around **navigating complex community dynamics**. Community voices can often be in opposition to each other, generating interpersonal conflicts. Powerful voices can claim to speak for a community and attempt to take ownership and control over community engagements, and our work must navigate these tensions with integrity. The second challenge involves **maintaining ethical and non-extractive community research engagements.** This includes the ongoing work of procuring grant funding to ensure that those we engage with are adequately remunerated for their labour, as a form of respect and reciprocity, and in recognition of their contributions to the *Walk With Me* project. It also includes the work of elevating the most vulnerable individuals in the communities we work with (often PWLLE), creating the conditions in which they can be supported as leaders, and valuing their voices in research as equal to, or above, those who possess the most power and the loudest voices. The third challenge is around the **resilience of oppressive systems.** We have noticed the remarkable ability of those in power to weaponize their authority, refusing to take responsibility for institutional and structural failings while shifting blame onto the most vulnerable in society. Even when introduced to new ways of working in community, institutional actors often continue to undertake the same problematic activities while attempting to sell solutions to the problems that they have created. We do not have one answer to address these challenges, but the first step involves slowing down, connecting with key allies, and having the wisdom to recognize historical complexities, power networks, community energies, and leadership directions that are embedded in place, even as we move forward.

Beyond these challenges, the *Walk With Me* project has also found exciting new opportunities. We have found that place-based teachings and practices have fostered a kind of collective wisdom, trust, and protection among our team that has allowed our CER project to overcome obstacles, grow, and expand while staying true to our core principles. As team member Caresse observed:

"Instead of blazing through the brick wall and knocking it down, [we] step back, group together and move around the wall, picking up everyone to come with us as we go. [...] [We're finding] another other way around, taking a step back, giving space and just finding a different direction, a different approach to [the problem] in a gentle way, in an ethical way, and a way that is inclusive and kind."

We also see the expansion of the project as a powerful opportunity to practice the circle and promote cultural safety with a wider public, drawing on the place-specific wisdom of knowledge-keepers in the territories where we walk, in the hopes of building deeper connections within and between communities attempting to address the TDPC. Perhaps most importantly, *Walk With Me* now has the opportunity to bring the stories and experiences of those on the front lines of the TDPC, those whose voices are most often suppressed or marginalized within quantitative research frameworks, to a wider audience. We aim to bring honour and respect to communities that have not gotten respect, working with them to forge a shared vision that can be communicated back to those with the power to make change.

In conclusion, our small CER project has made, and continues to make an outsized impact as we work in community to address the TDPC on Vancouver Island and beyond. We hold closely to the stories and voices of those most affected by the TDPC. Staying true to the core principles of CER and working with the place-based teachings of the circle, we continue to engage with Peers, allies, organizations, and governments using methods of cultural mapping. Though we have produced five major research reports to date, we still see our work in its infancy, with room for expansion beyond the geographies where we have been most active. It is our desire that the stories of those we walk with will continue to have impact and change the hearts and minds of those in power, fomenting empathy in a time where it is in such short supply. We also hope that other researchers will be able to learn from our work, to explore synergies, and break down silos between CER and biomedical research paradigms as we collectively respond to the TDPC and other complex crises in our communities. We aspire toward a more positive future: one in which stigma, violence, harm, and exclusion vanish, community bonds grow stronger, and preventable deaths from toxic drug poisoning become a thing of the past.

## Data Availability

The datasets generated and/or analyzed during the current study are not publicly available due to the possibility of individual privacy being compromised, in accordance with institutional ethics protocols.

## Abbreviations

BC: British Columbia
CER: Community Engaged Research
NIC: North Island College
PWLLE: People with Lived and Living Experience
SUS: Substance Use Support
TDPC: Toxic Drug Poisoning Crisis

## Footnotes

1 Our use of the term “toxic drug poisoning crisis” or TDPC, is reflective of a broader shift in language in British Columbia health research and in peer-reviewed literature, from terms like “overdose crisis” or “toxic drug crisis,” in response to insights from Indigenous peoples and people with lived and living experience. The language of TDPC highlights how the crisis is more than just accidental consumption of too much of a substance, rather, it represents a pervasive crisis of toxic drug poisoning stemming from an unregulated and tainted drug supply. Toxicity is occurring due to the lack of regulation, as well as the inability of users to consistently be informed of the substances they are ingesting, which are often poisonous [1–5].

2 British Columbia Coroner’s service data from 2023 shows that 87.6% of deaths from drug toxicity on Vancouver Island involved fentanyl and analogues, 48.6% involved benzodiazepines, and 50.5% involved methamphetamines (6). Likewise, 2023 data from The University of Victoria’s Substance Drug Checking lab (which provides free drug testing services on behalf of users), found similar results on Vancouver Island in 2023—with 85.4% of tested opioid samples containing fentanyl and analogues, 47.4% containing benzodiazepines, and 4.8% containing xylazine. Xylazine contamination has been decreasing on the Island overall since 2022, but remains very high in the Comox Valley, where it was found in 31.1% of samples checked in 2023 (13).

3 Smaller cities in BC's neighbouring jurisdictions being affected by similar substances, though to greater or lesser degrees. For example, in Whatcom County, Washington (containing the city of Bellingham, just south of the international border), 2022 data (the most recent available) shows fentanyl and analogues being found in 69% of toxic drug poisoning deaths. Meanwhile, in the smaller city of Lethbridge, Alberta, just east of the BC border, 96% of toxic drug poisoning deaths in 2023 (Jan-Nov) involved fentanyl and analogues, while only 6% involved benzodiazepines. Washington state does not include deaths involving benzodiazepines in their data (14,15).

4 Team member quotes, as well as the challenges and opportunities identified in the discussion section of this paper, are derived from a team reflection circle conducted on December 7, 2023.

5 Following Wallerstein et al. (2020), we view community-engaged research (CER) as an overarching term that can encompass closely related forms of research such as Community Based Participatory Research (CBPR) and Participatory Action Research (PAR). *Walk With Me* is grounded in ethical principles that intersect with all these research forms, and thus we use the term CER to describe our ongoing work.

6 In this paper, we use the inclusive terms “peers” and “people with lived and living experience (PWLLE)” rather than the more limited term “people who use drugs (PWUD),” to honour the diversity of knowledges and experiences among those with first-hand knowledge of the toxic drug poisoning crisis. The voices of PWLLE are central to *Walk With Me*’s work, and direct quotes from PWLLE can be found in of each of our published research reports (see 5, 21–24). In this article, we amplify the voices of *Walk With Me* team members with direct experience of our research process, including members who identify as PWLLE.

7 The initial project framework was developed by a team of researchers, community leaders, funders, and other stakeholders from the Comox Valley, Campbell River, and Kamloops, BC.

8 Recent data shows that the opioid supply in Campbell River is highly contaminated by benzodiazepines, more than any other community on Vancouver Island, appearing in 84.7% of all samples checked by the University of Victoria’s Substance Drug Checking lab in 2023 (13).
